# Initial Vestibular Function May Be Associated with Future Postural Instability in Parkinson’s Disease

**DOI:** 10.3390/jcm11195608

**Published:** 2022-09-23

**Authors:** Jeong Ho Park, Min Seung Kim, Suk Yun Kang

**Affiliations:** 1 Department of Neurology, College of Medicine, Soonchunhyang University Bucheon Hospital, Bucheon 14584, Gyeonggi-do, Korea; 2 Department of Neurology, Dongtan Sacred Heart Hospital, Hallym University College of Medicine, 7, Keunjaebong-gil, Hwaseong 18450, Gyeonggi-do, Korea

**Keywords:** Parkinson’s disease, postural instability, falls, vestibular-evoked myogenic potentials, predict, follow-up

## Abstract

Backgrounds: We aimed to understand the association between initial vestibular function examination and postural instability (PI) development in Parkinson’s disease (PD). Methods: After screening 51 PD patients, we divided 31 patients into 2 groups based on the presence of PI at the follow-up visit and compared the clinical features and vestibular-evoked myogenic potential (VEMP) variables. Results: The mean values of Hoehn and Yahr stage, Unified Parkinson’s Disease Rating Scale (UPDRS) part III, and item 30 (postural stability) of UPDRS were larger in patients with PI at a follow-up visit (*p* = 0.000, 0.006, 0.048, respectively). In VEMP analyses, the onset latencies of left and right cervical VEMPs were significantly reduced in patients with PI (*p* = 0.013, 0.040, respectively). Conclusion: We found that the initial VEMP test may be associated with later postural imbalance in PD, suggesting the baseline evaluation may help predict future PI occurrence. A more significant number of patients and more long-term follow-ups are likely to be required for confirmation.

## 1. Introduction

Postural instability (PI) is one of the most exhausting motor symptoms and a leading cause to fall in Parkinson’s disease (PD) [1,2]. PI is more frequent in PD with rapid progression and usually appears in advanced PD. Because PI is refractory to medical treatment, early recognition as possible and specific rehabilitative therapy may be necessary [3].

PI is associated with multiple factors, and its clinical features may be non-specific, preventing early detection and timely intervention [4]. The identification of measurable factors may be crucial in effective screening and monitoring PI development. Previously reported factors were fear of fall, biomechanical parameters (center of pressure, center of gravity, and center of mass), age, postural reflexes, defective perception of orientation, impulsivity, and serum vitamin D level [4]. Postural adjustments partly depend on the integrative sensory information from proprioceptive, visual, and vestibular functions [5]. The vestibular system, in particular, the vestibular nuclei in the brainstem, plays an essential part in the integrative sensory process [6], and vestibular dysfunction is thought to be involved in PD [5,7].

A recent study suggested that the abnormal vestibular system might predict increased fall incidences in PD with postural imbalance [8]. However, they include patients with falling and complete postural imbalance (i.e., the patient would have fallen if the examiner had not caught the patient during the fall in the pull-test). The studied groups were PD, and atypical parkinsonism analyzed together. There were also studies showing that vestibular dysfunction could not explain PI [9]. The vestibular system incorporates various sensory information and outgoing motor adjustments to maintain balance, and PI is associated with the sensory input and motor coordination. We hypothesized vestibular dysfunction would contribute to the development of PI and would be different between patients with and without future PI development. If so, the measurement of the vestibular system could be a biomarker to predict the occurrence of PI in PD not having falls and complete postural imbalance. The vestibular-evoked myogenic potential test (VEMP) is a detailed electrophysiological test that evaluates the vestibular system [10]. We investigated whether the initial VEMP measurements differed between PD having later PI and not having later PI. More specifically, PD with late PI would show more impaired vestibular function. We assumed that the VEMP response would be delayed or reduced.

## 2. Materials and Methods

### 2.1. Patients

**Figure 1:**
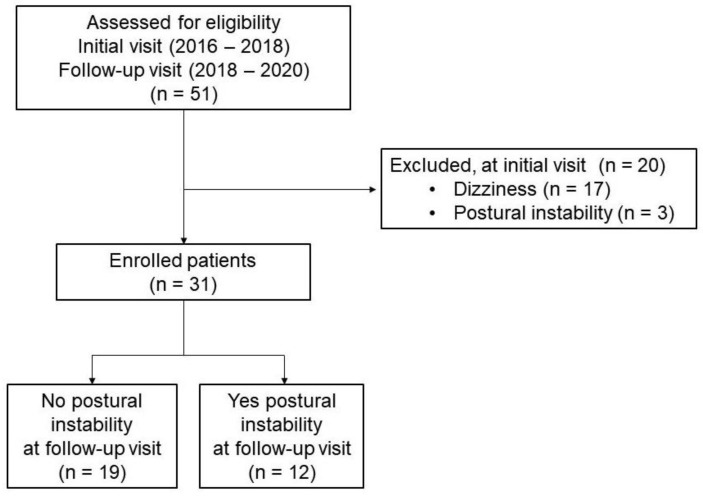
Patient disposition.

We initially screened 51 PD patients who underwent clinical evaluations and VEMP at the first visit (February 2016 to January 2018) and clinical evaluations at the follow-up visit (April 2018 to February 2021). PD was diagnosed according to UK Brain Bank criteria [11]. The clinical evaluations were Hoehn and Yahr stage (H&Y stage), the Unified Parkinson’s Disease Rating Scale (UPDRS) part III, disease duration, the Korean version of the Mini-Mental State Examination (K-MMSE), education, total levodopa equivalent daily dose (LEDD), the presence of orthostatic hypotension, and the presence of dizziness. To assess posture, gait, and balance, we used the following items of UPDRS part III: 27 (arising from a chair), 28 (posture), 29 (gait), and 30 (postural stability). Orthostatic hypotension follows the consensus definition of a sustained reduction in systolic blood pressure of at least 20 mmHg or diastolic blood pressure of 10 mmHg within 3 min of standing or head-up tilt to at least 60° on a tilt table [12]. Dizziness was chronic for over three months without vestibular dysfunction and complete postural imbalance [9]. The interval between initial and follow-up visits was 28.3 ± 8.5 months. We excluded 20 patients with dizziness or definite postural instability (i.e., absence of postural response; would fall if not seized by the examiner) at the first visit. Finally, 31 patients were enrolled (Figure 1). We divided the patients into two groups according to postural instability (PI) on neurological examination at the follow-up visit and compared the clinical symptoms and VEMP variables of the initial visit. The presence of PI was determined by item 30 (more than one = retropulsion, but recovers unaided). We also compared clinical features at follow-up. The study was approved by our Institutional Review Board of Soonchunhyang University Bucheon Hospital (IRB Number: SCHBC 2020-12-028-001). All methods were carried out in accordance with relevant guidelines and regulations. Informed consent was obtained from all participants and/or their legal guardians. This study was performed in accordance with the Declaration of Helsinki. The study was conducted in accordance with good clinical practice.

### 2.2. VEMP Recordings and Measurements

The methods of the VEMP recordings and measurement were previously described in detail [9]. We record all VEMPs with disposable silver/silver-chloride surface electrodes.

For ocular VEMP (oVEMP) recording, we placed the active electrodes symmetrically over the middle part of the lower eyelids, on top of the inferior orbital edges, and the reference electrodes 2 cm below these. During the recording, we requested the participants to sit upright and look upward at a fixed target (upward eye deviation of about 30°). We checked the peak latencies of the N1 and P1 and the N1-P1 peak-to-peak amplitudes.

For cervical VEMP (cVEMP) recording, we placed the active electrodes symmetrically over the upper middle part of the sternocleidomastoid muscle bellies with the reference electrode over the sternal manubrium. We requested the patients to lift their heads up from a headrest and turn their heads away from the ear that was being stimulated. We evaluated the peak latencies of the P13 and N23 and the P13-N23 peak-to-peak amplitude.

The oVEMPs and cVEMPs were elicited acoustically by employing short tone bursts with an acoustically shielded headphone (Telephonics TDH-39P, Welch Allyn, Inc., Middleton, WI, USA; 2 ms rise/fall and 2 ms plateau, frequency 500 Hz, 105 dB nHL). We used a VikingQuest EMG system (VIASYS Healthcare Inc., Middleton, WI, USA) for oVEMP and cVEMP recordings. All evoked responses were amplified (5000×), band-pass filtered (30–1500 Hz), notch filtered, averaged, and recorded without artifact rejection. We averaged 120 acoustic stimuli for each trial and performed three reproducibility trials.

### 2.3. Statistical Analysis

Data are expressed as means ±  SD and frequency. Demographic and clinical variables and VEMP parameters were compared between patients with and without PI. The statistical significance of the demographic and clinical variables was evaluated by the Mann–Whitney, Chi-square test, or Fisher’s exact test, where appropriate. We provided the effect size of differences in the two groups using Cohen d and the 95% confidence interval (CI) for the significant results. If we could not obtain VEMPs, they were recorded as “bilateral absent” or “unilateral absent”, and the ratio of the three conditions (i.e., bilateral absent, unilateral absent, and present) was compared. Values of *p*  <  0.05 were regarded as significant. Statistical analysis was performed using IBM SPSS 27 Statistics (IBM Corp., Armonk, NY, USA).

## 3. Results

Clinical features and VEMP values between patients without and with PI are summarized in Table 1 and Table 2. In initial clinical features, the mean values of HY stage, UPDRS III, and item 30 were higher in patients with PI at a follow-up visit (Table 1, *p* = 0.000, 0.006, 0.048, respectively; Cohen’s d = 0.5839, 7.6034, 0.4332; 95% CI = −2.551 to −0.860, −1.696 to −0.172, −1.740 to −0.209). In VEMP measurements, there were no differences in oVEMP parameters between the two groups (all *p*’s > 0.1), but N23 latencies of left and right cVEMPs were significantly shorter in patients with PI than in patients without PI (Table 2, *p* = 0.013, 0.040, respectively; Cohen’s d = 3.6169, 3.9766; 95% CI = 0.015 to 1.594, −0.011 to 1.613). For other parameters, the mean values of latency and amplitudes were small in patients with PI, but there was no statistical difference between the two groups (all *p*’s > 0.1). In follow-up evaluation, in addition to HY stage (*p* = 0.000; Cohen’s d = 0.3448; 95% CI = −3.744 to −1.726) and UPDRS III (*p* = 0.016, Cohen’s d = 6.6473; 95% CI = −1.785 to −0.247), item 27, 28, and 29 scores were statistically higher in the patients in PI group (Table 1, *p* = 0.012, 0.000, 0.002, respectively; Cohen’s d = 0.3650, 0.3650, 0.3732; 95% CI = −2.257 to −0.631, −3.298 to −1.411, −2.564 to −0.869).

The ratios of the bilateral absence, unilateral absence, and presence of all VEMPs were not different between patients without and with PI (all *p*’s > 0.2, Supplementary Table S1).

## 4. Discussion

Our study shows that VEMP values may differ between patients who later develop PI and those who do not, suggesting predicting the occurrence of PI in patients without PI. Among patients who initially did not have dizziness and PI, those who developed later had initial different VEMP findings from those who later did not develop PI. The variables showing statistical differences were the N23 latencies of bilateral cVEMPs. Although there was no statistical difference in all variables, overall latencies were short, and amplitudes were low in patients with PI.

It seems that cVEMP may be suitable for predicting future PI in our study. The anatomical pathways of the oVEMP and cVEMPs are thought to be different: an acceding pathway at the upper pons and midbrain level in oVEMP and a descending pathway from the vestibular nucleus in cVEMP [9]. However, since some previous studies also reported abnormal response of oVEMP in PD, this needs more investigation [10,13].

The shorter latency in patients with PI than in patients without PI may be unexpected findings in our study. Although a recent study showed short latency of cVEMP in PD compared to controls [9], several prior investigations reported delayed latencies and low amplitude in PD compared to controls. We do not know the exact reason, but one possible explanation is due to the compensatory mechanism because we only included the early stage of PD [9]. Although direct comparisons with initial VEMP findings were difficult because bone-conducted vibration stimuli such as tendon hammer taps were applied at the follow-up visit, the latencies of oVEMP were longer in PD without PI, and the latencies of cVEMP were shorter (Supplementary Table S2).

There were no studies about the relationships between initial VEMP findings and later PI occurrence in PD and VEMP measurements as predictors of PI. A recent study reported that VEMP findings could indicate future falling in patients with PD and atypical parkinsonism [8]. Although the study design differed from ours (i.e., telephone interviews to check fall occurrence), we think our findings support the study, because PI is one of the specific PD-related fall risk factors [14].

Our study has several limitations. First, the sample size was small and was not calculated in this study. We acknowledge it might influence the results. This issue is relevant when arguing that VEMPs may be considered a predictive factor for PI development. Of course, studies with more patients are needed, but we think this study has suggested a new clinical value of VEMP. Second, the follow-up period of about two years might be a little short to observe the occurrence of PI in PD. Third, some initial clinical features (i.e., HY stage, UPDRS III) were not similar between groups without PI and with PI, maybe because we only excluded patients with clear postural imbalance. These factors might affect our VEMPs results. Forth, there was not any comparison between data at baseline and data at follow-up, and if performed, these comparisons would have been clinically useful. Unfortunately, we could not, because the VEMP recording techniques were different as we mentioned before. Fifth, we did not include healthy controls. In order to be clinically useful, a comparative study with healthy control seems to be necessary. Sixth, we did not perform other sophisticated instrumental assessments of vestibular function, including caloric tests, electro- or video nystagmography, dynamic visual acuity, and posturography [10,15]. A more significant number of patients, more long-term follow-up, and more detailed vestibular evaluation are likely to be warranted to confirm our observations. Seventh, it can be argued that the posture in PD could affect VEMP findings [16,17,18] because PD patients usually have abnormal posture such as stooped or bent. Although we did not perform detailed objective measurements for posture, the item 28 score (i.e., posture) between PD without PI and with PI was similar.

## 5. Conclusions

In conclusion, our study suggests that VEMP test can be used to predict future PI, which requires further investigation with larger sample size. Early detection would be helpful by providing early interventions, such as rehabilitation, for PD patients at high risk of developing PI.

## Figures and Tables

**Table 1:** Clinical characteristics of patients without postural instability and with postural instability.

			No Postural Instability *n* = 19	Yes Postural Instability *n* = 12	*p* *-Value
Clinical Characteristics					
Initial Visit					
	Age, years		60.6 ± 7.6	62.9 ± 9.8	0.459
	Women, *n* (%)		12 (63.2)	4 (33.3)	0.106
	Duration of disease, years		2.5 ± 1.2	3.8 ± 2.7	0.326
	K-MMSE		26.9 ± 3.6	26.3 ± 2.7	0.326
	Education, years		9.5 ± 5.0	11.5 ± 4.1	0.328
	Orthostatic hypotension, *n* (%)				0.280
		not checked	4 (21.1)	2 (16.7)	
		no	14 (73.7)	7 (58.3)	
		yes	1 (5.3)	3 (25.0)	
	HY stage		1.3 ± 0.7	2.3 ± 0.3	0.000
	UPDRS III		12.8 ± 8.3	20.0 ± 6.3	0.006
		Item 27	0.1 ± 0.3	0.1 ± 0.3	0.921
		Item 28	0.2 ± 0.4	0.5 ± 0.5	0.191
		Item 29	0.1 ± 0.3	0.4 ± 0.5	0.152
		Item 30	0.2 ± 0.4	0.6 ± 0.5	0.048
	Total LEDD		399.9 ± 210.8	424.8 ± 293.1	0.120
Follow-up visit					
	K-MMSE		26.8 ± 3.6	25.7 ± 3.9	0.509
	HY stage		1.6 ± 0.4	2.5 ± 0.0	0.000
	UPDRS III		12.5 ± 6.4	19.3 ± 7.1	0.016
		Item 27	0.1 ± 0.2	0.6 ± 0.5	0.012
		Item 28	0.1 ± 0.2	0.9 ± 0.5	0.000
		Item 29	0.1 ± 0.3	0.8 ± 0.5	0.002
		Item 30	0.0 ± 0.0	1.0 ± 0.0	-
	Total LEDD		439.0 ± 174.5	507.3 ± 262.8	0.509
	Dizziness severity		0.3 ± 0.5	0.5 ± 0.5	0.412
Interval between initial and follow-up visit, months			30.0 ± 8.6	25.7 ± 8.1	0.191

Values are expressed as means ± SD or number (percentage). Dizziness severity was evaluated with visual analogue scale having a range of scores from 0 to 10. Item 27–30 of UPDRS III are assessments of arising from chair, posture, gait, and postural stability, respectively. * *p* < 0.05 indicates significant differences. Abbreviations: K-MMSE, the Korean version of the Mini-Mental State Examination; HY stage, Hoehn and Yahr stage; UPDRS III, Unified Parkinson’s Disease Rating Scale part III; Total LEDD, total levodopa equivalent daily dose.

**Table 2:** Initial VEMP parameters of patients without postural instability and with postural instability.

			No Postural Instability *n* = 19	Yes Postural Instability *n* = 12	*p* *-Value
VEMP Parameters of Initial Visit					
	oVEMP				
	Left				
		N1 latency (ms)	12.7 ± 1.2	12.4 ± 2.4	0.733
		P1 latency (ms)	17.3 ± 1.1	15.6 ± 2.7	0.186
		N1-P1 amplitude (μV)	2.4 ± 3.0	1.6 ± 1.1	0.795
	Right				
		N1 latency (ms)	13.0 ± 1.6	13.0 ± 2.7	0.979
		P1 latency (ms)	17.4 ± 1.1	16.2 ± 2.8	0.373
		N1-P1 amplitude (μV)	1.9 ± 1.6	1.4 ± 0.8	0.459
	cVEMP				
	Left				
		P13 latency (ms)	15.8 ± 3.8	14.7 ± 2.3	0.458
		N23 latency (ms)	24.0 ± 4.4	21.0 ± 1.7	0.013
		P13-N23 amplitude (μV)	123.4 ± 157.2	82.3 ± 61.5	0.412
	Right				
		P13 latency (ms)	16.2 ± 36.5	15.0 ± 2.0	0.604
		N23 latency (ms)	24.5 ± 4.7	21.3 ± 2.0	0.040
		P13-N23 amplitude (μV)	116.9 ± 98.5	72.2 ± 67.3	0.152

Values are expressed as means ± SD. * *p* < 0.05 indicates significant differences. Abbreviations: VEMP, vestibular-evoked myogenic potential test; oVEMP, ocular VEMP; cVEMP, cervical VEMP.

## Supplementary Materials

The following supporting information can be downloaded at: https://www.mdpi.com/article/10.3390/jcm11195608/s1, Table S1: Presence of VEMP responses of patients without postural instability and with postural instability; Table S2: Follow-up VEMP findings of patients without postural instability and with postural instability.
