# Supplementary material for: Initial Vestibular Function May Be Associated with Future Postural Instability in Parkinson’s Disease
Source: J Clin Med. 2022 Sep 23;11(19):5608. doi: 10.3390/jcm11195608 (PMC9570519; doi:10.3390/jcm11195608)
Supplement: Supplementary file 1 [file jcm-11-05608-s001.zip › jcm-1918385-supplementary.pdf]

Journal of
Clinical Medicine

MDPI

Supplementary Table S1. Presence of VEMP responses of patients without postural instability and with postural instability.

	No Postural Instability $n = 19$	Yes Postural Instability $n = 12$	p *-Value
**Initial visit**			
oVEMP, n (%)			0.229
bilateral absent	4 (21.1)	0 (0.0)	
unilateral absent	3 (15.8)	2 (16.7)	
present	12 (63.2)	10 (83.3)	
cVEMP, n (%)			0.930
bilateral absent	1 (5.3)	1 (8.3)	
unilateral absent	2 (10.5)	1 (8.3)	
present	16 (84.2)	10 (83.3)	

* $p < 0.05$ indicates significant differences.
Abbreviations: VEMP, vestibular evoked myogenic potential test; oVEMP, ocular VEMP; cVEMP, cervical VEMP.

Supplementary Table S2. Follow-up VEMP findings of patients without postural instability and with postural instability.

	No Postural Instability $n = 19$	Yes Postural Instability $n = 12$	p *-Value
**Parameters**			
oVEMP			
Left			
N1 latency (ms)	6.6 ± 2.0	6.9 ± 0.7	0.023
P1 latency (ms)	11.1 ± 1.9	20.3 ± 28.8	0.052
N1-P1 amplitude (μV)	10.2 ± 6.1	9.2 ± 6.8	0.509
Right			
N1 latency (ms)	6.5 ± 2.1	6.4 ± 1.0	0.372
P1 latency (ms)	10.9 ± 2.0	11.1 ± 1.1	0.372
N1-P1 amplitude (μV)	11.2 ± 6.2	9.1 ± 6.1	0.367
cVEMP			
Left			
P13 latency (ms)	26.0 ± 41.9	13.2 ± 2.2	0.000
N23 latency (ms)	25.3 ± 2.9	22.2 ± 3.4	0.006
P13-N23 amplitude (μV)	153.7 ± 123.2	124.9 ± 36.5	0.984
Right			
P13 latency (ms)	15.7 ± 2.6	14.1 ± 2.8	0.110
N23 latency (ms)	25.3 ± 2.8	24.1 ± 4.2	0.389
P13-N23 amplitude (μV)	148.6 ± 104.7	108.5 ± 36.2	0.101
Presence of VEMP responses			
oVEMP			0.142
bilateral absent	0 (0.0)	0 (0.0)	
unilateral absent	0 (0.0)	2 (16.7)	
present	19 (100.0)	10 (83.3)	
cVEMP			-
bilateral absent	0 (0.0)	0 (0.0)	
unilateral absent	0 (0.0)	0 (0.0)	
present	19 (100.0)	12 (100.0)	

Values are expressed as means ± SD or number (percentage).
* $p < 0.05$ indicates significant differences.
Abbreviations: VEMP, vestibular evoked myogenic potential test; oVEMP, ocular VEMP; cVEMP, cervical VEMP.
